# Top-down structuring of freshwater bacterial communities by mixotrophic flagellates

**DOI:** 10.1038/s43705-023-00289-7

**Published:** 2023-09-02

**Authors:** Marina Ivanković, Robert Ptacnik, Mia Maria Bengtsson

**Affiliations:** 1 WasserCluster Lunz - Biologische Station GmbH, Lunz am See, Austria; 2 Department of Functional and Evolutionary Ecology, University of Vienna, Vienna, Austria; 3 Institute of Microbiology, University of Greifswald, Greifswald, Germany

**Keywords:** Water microbiology, Freshwater ecology, Microbial ecology, Microbial ecology

## Abstract

Mixotrophic and heterotrophic protists hold a key position in aquatic microbial food webs. Whereas they can account for the bulk of bacterivory in pelagic systems, the potential structuring effect of these consumers on bacterial communities is far from clear. We conducted short-term grazing experiments to test for the overall impact on bacterial community structure and possible prey preferences of phagotrophic protists. The protist taxa selected for this study include three mixotrophic flagellates, comprising two obligate- and one facultative mixotroph, and one phagoheterotrophic flagellate lacking phototrophic capacity. Bacterioplankton from seven different lakes were enriched and used to represent semi-natural prey communities. Our study demonstrated protist strain specific impacts on bacterial community composition linked to grazing. The three mixotrophs had variable impacts on bacterial communities where the two obligate mixotrophs exhibited lower grazing rates, while showing a tendency to promote higher bacterial diversity. The phagoheterotroph displayed the highest grazing rates and structured the bacterial communities via apparent selective grazing. Consistent selectivity trends were observed throughout the experiments, such as the apparent avoidance of all flagellates of *Actinobacteria*, and high grazing on dominant *Burkholderiales* taxa. However, there was no consistent “fingerprint” of mixotrophic grazing on prey communities, but the structuring impact rather seemed to depend on the trophic mode of the individual protist taxa, i.e. their dependence on phototrophy vs. phagotrophy. Our findings highlight the differential structuring impact of protist taxa on bacterial communities which may have important ecological implications, for example during periodic dominance of obligate mixotrophic bacterivores in changing lake ecosystems.

## Introduction

Mixotrophic and heterotrophic protists are recognized as key consumers of bacteria in the pelagial, thereby themselves holding a major position in aquatic food webs and overall pelagic ecosystem functioning [1–3]. Protist bacterivory not only controls the abundance and biomass of bacterial populations, it also has a profound impact on bacterial community structure (see [3, 4], and references therein). Mixotrophic bacterivory is a common trait in freshwater and marine plankton, often of at least equal importance in the removal of various picoplanktonic cells as predation by heterotrophic bacterivores [5–7]. Currently, the understanding of the role of mixotrophic protists in aquatic food webs is hampered by a lack of data on their impact on both lower and higher trophic levels [8].

Organisms combining an autotrophic and heterotrophic nutrition are considered to be mixotrophic [7]. When addressing predatory protists, the term mixotrophy refers to protists combining phototrophy with phagotrophic uptake of prey [9]. This dual strategy enables mixotrophic protists to cover demands for nutrient and energy from alternative sources. They can utilize essential nutrients from ingested prey and from the surrounding water, while covering necessary energy and carbon demands over photosynthesis and prey ingestion [10]. The variety of empirical observations and described mixotrophic taxa are suggesting that the mixotrophic nutrition is covering a gradient between clear heterotrophic and phototrophic nutrition [10]. Different mixotrophic taxa can cover a wider or narrower range between these two nutritional strategies, by changing their phototrophic:phagotrophic lifestyle ratio in relation to the existing environmental conditions [1, 11, 12]. Simple functional classifications often reflect the main nutritional strategy of mixotrophic taxa, resulting with two main groups, obligate (OM) and facultative mixotrophs (FM) [1, 13]. Growth of FM is primarily heterotrophic, mainly depending on prey supply, with light and inorganic nutrients often serving as minor resources [14–16]. Conversely, OM taxa are primarily phototrophic, mainly rely on photosynthesis, and ingest lesser amounts of bacteria often depending on the availability of dissolved nutrients and light [16–18]. The quantitative dependence of these two mixotrophic groups on bacterial prey is relatively well known, in contrast to the influence they have on bacterial community composition (BCC).

Protists predation is well known to induce shifts in bacterial communities via selective grazing [19–22]. For example, prey cell size is an important factor for selectivity, which is often linked to the specific morphology and feeding strategy of the predator. In addition, different anti-predator traits such as aggregate formation allow bacteria able to escape grazing and persist in the environment. Further, some predators apparently employ more sophisticated selection mechanisms resulting in egestion of non-preferred ingested prey (selective digestion [23]). Although such selective feeding likely reflects nutritional needs tightly linked to the physiology of the predator, their consequences for structuring prey communities are not well understood. Moreover, most studies addressing these structuring shifts in bacterial communities focused on heterotrophic protists, while if mixotrophic protists were considered, the focus was mainly on FM with relatively high growth and grazing rates. Studies addressing selectivity of mixotrophs typically used model prey such as selected cultured strains, fluorescently labeled bacteria, or fluorescent beads (e.g. refs. [6, 23]). However, the impact of mixotrophic predators on the diverse microbial communities typically found in lakes and oceans has rarely been considered. For example, the relatively more complex physiology of mixotrophs may translate into different nutritional needs and thereby selectivity for certain bacterial prey taxa. The interaction of mixotrophs with bacteria is further complicated by their photosynthetic activity, which may lead to stimulation of bacterial growth through exudation.

In order to investigate the impact of mixotrophic and heterotrophic bacterivores on diverse bacterial prey communities, we conducted controlled short-term grazing experiments where we exposed variable bacterial communities from different lakes to selected flagellate bacterivores belonging to *Chrysophyceae*. The employed protist taxa exemplify different trophic modes, with two OM taxa employing phototrophy as the dominant strategy, a FM relying more on phagotrophy, and one phagoheterotroph lacking phototrophic capacity. Natural plankton communities from seven different lakes were collected and enriched to create a variation of diverse semi-natural bacterial communities serving as prey. We hypothesized that the selected protists would impact bacterial community composition through their grazing activity. Specifically, we hypothesized that 1) BCC change will mainly depend on the grazing rate of the protists, with higher bacterial mortality leading to larger changes in BCC; 2) higher bacterial mortality will lead to a decrease in bacterial diversity due to the accumulation of grazing-resistant bacterial taxa which are avoided by the protists and therefore become dominant. Further, we aimed to identify general patterns in prey selectivity of the individual protists that are consistent across variable bacterial prey communities.

## Material and methods

### Experimental organisms

Four bacterivorous protist taxa were employed as bacterivores, three mixotrophic flagellates: *Uroglenopsis americana*, *Ochromonas* c.f. *perlata* and *Poterioochromonas malhamensis*, and one heterotrophic *Spumella*-like flagellate. The protists are hereinafter referred to with their genus name. These flagellates are taxonomically closely related and similar in their cell morphology (apart from colonial growth in *Uroglenopsis*), but represent different trophic modes, from primarily phototrophic OM (*Uroglenopsis, Ochromonas*) to a primarily phagotrophic FM (*Poterioochromonas*) and a phagoheterotroph lacking phototrophic capacity entirely (*Spumella*). For a detailed description addressing the protists see Table 1. The prey communities originate from seven different natural lakes in Austria and south-eastern Bavaria: Lunzer See, Mittersee, Obersee, Erlaufsee, Hubertussee, Klostersee and Chiemsee (see Supplementary Table S1 for lake descriptions). In order to test for general trends in terms of protist-bacteria interactions, the chosen lakes represent a range of different potential habitats for the consumers employed in this study.

**Table 1:** Characteristics of protist taxa used as bacterial consumers.

Taxon	Nutrition	Class	Shape	Colony forming	Average cell volume (µm³) ± SD	Origin	Strain ID	Reference
*Uroglenopsis americana*	Obligate mixotrophy	Chrysophyceae	Tear-drop	Yes	164.33 ± 52.48	Lunzer See, Austria	Own isolate	This study
*Ochromonas* cf. *perlata*	Obligate mixotrophy	Chrysophyceae	Spherical	No	146.73 ± 38.84	Lunzer See, Austria	Own isolate	−16
*Poterioochromonas malhamensis*	Facultative mixotrophy	Chrysophyceae	Spherical	No	165.54 ± 87.39	Lake Constance, Germany	933–1a, 933–1c, ’DS’	[70]
*Spumella* sp.	Heterotrophy	Chrysophyceae	Spherical	No	88.28 ± 16.54	Lunzer See, Austria	Own isolate	This study

### Cultivation and preparation of prey bacteria and protist prior to the experiment

All organisms were pre-cultivated in autoclaved glass bottles placed in a walk-in environmental chamber, at a constant temperature of 18 °C and a light:dark cycle of 16:8 h, with a smooth transition between light and dark phases. The light source consisted of three different LEDs, mimicking natural PAR, supplying non-limiting irradiance at ca. 80 µmol m^−2^ s^−1^. The handling of the lake prey communities, as well of the protist cultures prior and during the experiment was done under a laminar flow hood under sterile conditions.

Bacterial communities were sampled from the epilimnion of seven natural lakes. On a given lake we collected a 10 L water sample with a clean container, rinsed with lake water. The water was immediately transported to the laboratory under dark and cooled conditions and vacuum filtered (200 mbar) through 0.8 μm polycarbonate membrane filters to exclude protists and any larger organisms. In order to create the same growing conditions for all bacterial prey communities during the cultivation phase the filtrate was pelleted (3000 G, 10 min, RT) and resuspended in >10 weeks aged 0.2 µm sterile filtered Lunzer See water. Organic carbon and inorganic phosphorus were added according to the Redfield ratio (Glucose, 83 µmol C L^−1^, Monopotassium phosphate, 0.79 µmol P L^−1^) to support bacterial growth. No nitrogen source was added, as nitrogen is a non-limiting nutrient in the Lunzer See [24]. The prey communities were kept in exponential growth for 48 h in the enriched medium until further processing. Bacterial prey was added to the experimental incubations at a cell density of 3.3–6.6 million cells ml^−1^. The protist cultures were pre-grown on medium based on sterile filtered lake water (same as used for bacteria) with a 5% final addition of modified WEES medium (WEES Medium Recipe v.03.2007 without soil extract [25]. Protists were kept in exponential growth prior to every experiment. Protists were added to the experimental incubations at a cell density of 4060–12,600 cells ml^−1^.

In order not to transfer P-rich growth medium together with the pre-grown bacteria or protists into the experimental incubations, the pre-grown bacteria and protist cells were separated from the culture medium. All bacterial prey communities and protist predators, except *Uroglenopsis*, were pelleted via centrifugation (3000 G, 10 min, RT), and subsequently resuspended in aged sterile 0.2 µm filtered Lunzer See water. *Uroglenopsis* colonies are highly sensitive to mechanical stress and were therefore concentrated via light attraction in a large, graduated cylinder, carefully collected with a pipette near the surface and resuspended. After resuspension the cultures were kept undisturbed for 24 h to adapt to the sterile lake water and to recover from any stress induced by centrifugation or pipetting (the used recovery time turned out to prevent negative effects on flagellate growth, tested in preliminary experiments). The final experimental incubations were done in aged sterile 0.2 µm filtered Lunzer See, without nutrient addition. Lunzer See water was chosen to achieve low P-concentrations in the experimental incubations, as P-limitation may trigger phagotrophy in mixotrophs [18, 26]. The Lunzer See is an oligotrophic lake, with orthophosphate levels typically well below 2 µg L^−1^ during summer stratification (i.e. time of water sampling, Preiler & Ptacnik, unpublished data, ongoing monitoring program). Due to low densities during the pre-cultivation, *Ochromonas* was not employed in the Lunzer See and Obersee experiments. The protist cultures used in this study were not axenic, i.e. they contained heterotrophic bacteria.

### Experimental setup, incubation and sample processing

The natural lake communities were collected to provide an array of semi-natural, diverse bacterial prey communities. After the pre-cultivation and recovery phase, the prey community from a given lake was separately inoculated with each protist (‘Protist treatment’), with one control treatment containing the bacterial prey only (‘Control’). Since the protist cultures were not axenic, bacteria were introduced along with the protist culture, specific for every protist treatment, but not present in the Control, further on called ‘Background bacteria’. Per lake and treatment, a volume of 2 L was prepared, gently shaken and split into four 500 ml autoclaved glass bottles. One bottle was immediately harvested (‘Start’ of experiment), the remaining three bottles were incubated for 48 h and harvested (‘End’ of experiment). The experimental incubations were carried out under identical conditions as described above for the pre-cultivation phase. From every bottle, samples were collected for flow cytometry, microscopic and molecular analyses. Samples for flow cytometry were fixed with a mixture of 0.2 µm filtered paraformaldehyde and glutaraldehyde with a final concentration of 0.01% and 0.1%, respectively, in the sample, and subsequently stored at 4 °C until analysis (within 24 h after fixation). A volume of 400 ml was filtered on polyethersulfone 0.2 μm membrane filters and kept frozen at −80 °C until DNA extraction.

### Estimation of growth and mortality rates

Cell numbers were estimated via flow cytometry using a Beckman Coulter CytoFLEX flow cytometer (Beckman Coulter GmbH, Krefeld, Germany), data acquisition and analysis were carried out using the CytExpert Software 2.3. To count heterotrophic organisms, subsamples were stained with a SYTO 13 (Invitrogen, final concentration 0.5 μM). After stain addition, the sample was kept 15 min at RT in the dark and subsequently analyzed via flow cytometry as described below. Stained bacteria and *Spumella* cells were gated on a dot plot of blue absorption light and green fluorescence versus the forward scatter. Using unstained samples, the mixotrophs were gated by their Chl-*a* auto-fluorescence signal by using a scatter plot of blue excitation light and red fluorescence light versus the forward scatter. The thresholds were set to minimize background noise, and the organisms were gated manually.

Net growth rates of bacteria and protists in the incubation bottles were calculated for each treatment replicate as:

$${{{\mathrm{Net}}}}\,{{{\mathrm{growth}}}}\,{{{\mathrm{rates}}}}\,\mu = \left( {\ln \left( {{{{\mathrm{N}}}}_{{{{\mathrm{End}}}}}} \right) - \ln \left( {{{{\mathrm{N}}}}_{{{{\mathrm{Start}}}}}} \right)} \right)/\left( {{{{\mathrm{t}}}}_{{{{\mathrm{End}}}}} - {{{\mathrm{t}}}}_{{{{\mathrm{Start}}}}}} \right)$$

where N is the estimated cell abundance at the experimental start and end. Bacterial mortality was estimated as:

$${{{\mathrm{Mortality}}}} = \mu _{{{{\mathrm{Control}}}}} - \mu _{{{{\mathrm{Treatment}}}}}$$

where *μ*_Control_ is the average bacterial growth rate in the Controls and *μ*_Treatment_ is the bacterial growth rate in the corresponding protist treatment replicate.

### DNA extraction, Illumina amplicon sequencing and bioinformatic analyses

Total community DNA was extracted from the filters using the DNeasy PowerSoil Kit (QIAGEN, Hilden, Germany) according to the manufacturer´s instructions with minor modifications in the lysis step; mechanical lysis was achieved by bead-beating pre-cut filters in a Retsch MM2 swing mill (Retsch GmbH, Haan, Germany) for 5 min at a rate of 70 strokes per second. Extracted DNA was amplified with primer pairs targeting the V4 region of the 16S rRNA gene (515f: 50-GTGYC AGCMGCCGCGGTAA-30, 806r: 50-GGACTGCNVGGGTWTC TAAT-30 [27], coupled to custom adaptor-barcode constructs. PCR amplification and Illumina MiSeq library preparation were carried out by LGC Genomics (Berlin, Germany). Adaptor and primer clipped sequences were processed and denoised using the DADA2 pipeline (v.1.2.0 [28] using R (v.4.1.2 [29]). All forward and reverse Illumina reads were simultaneously trimmed to 200 bp and filtered out if the quality threshold was not met (maxEE = 2, minLen = 175). The filtered sequences were then de-replicated and error rates were used to infer sample sequences into amplicon sequence variants (ASVs). Paired forward and reverse sequence reads were merged and followed by a chimera sequence check and removal. Sequences have been submitted to the NCBI short read archive (bioproject PRJNA863089). The resulting ASVs were used to construct a table containing relative abundances of ASVs across all samples. The Silva search and classify function in combination with the Silva database (v.138.1 [30]) was used to classify ASVs to taxa at the lowest taxonomical levels possible. Further, ASVs assigned to chloroplast or mitochondria and ASVs read counts contributing less than 0.01% to the overall library size were excluded. Background bacteria ASVs were defined as those ASVs present in the respective protist samples (protist culture combined with prey bacteria), but not in the Control samples (prey only) at the experimental start. As the scope of this study is understanding the impact of the selected protist on different bacterial communities originating from natural lake communities, we excluded background bacteria ASVs from the dataset by subsequently subtracting them from all samples. 16S rRNA gene copy number (GCN) was estimated as a functional trait proxy for bacterial growth strategy, assuming that bacterial taxa with high 16S rRNA GCN have a higher potential growth rate owing to rapid expression of ribosomes [31, 32]. GCN was estimated per ASV based on the rrnDB (v.5.7 [33]).

### Statistical analyses

ASV richness was calculated by rarefying the whole dataset read counts to the lowest number of reads in a sample. Pielou’s evenness was calculated as H’/ln (rarefied richness), where H’ is the Shannon diversity index [34]. The changes in richness and evenness were calculated as the difference between end and start values for the parameter in question. Significant differences between treatments were evaluated per experiment by a Kruskal–Wallis test. Multiple comparisons where followed by a Dunn’s post hoc test, including a Bonferroni’s correction. Multivariate statistical analyses were carried out using the vegan package (v.2.6.2 [35]). Non-metric multidimensional scaling was performed on Hellinger-transformed sequence counts using Bray–Curtis dissimilarity to visualize similarities in ASV composition between samples. Bacterial community turnover was quantified by Bray–Curtis dissimilarity calculated between start and time points per sample. Spearman’s rank correlation analysis was used to assess the correlations between protist growth, bacterial mortality, bacterial community turnover, changes in bacterial richness and evenness. PERMANOVA was used to evaluate variation in community composition in response to lake and treatment. Differential abundance analyses were used as implemented in the DEseq2 R package (v.1.36.0 [36]) to identify differently abundant ASVs between Control and protist treatments per lake using raw sequence counts of all ASVs (i.e. without excluding ASVs). All statistical analyses were performed in R (v.4.1.2 [29]). Figures were generated using the ggplot2 package (v.3.3.6 [37]).

## Results

### Bacterial community composition

Illumina amplicon sequencing of 16S rRNA gene fragments resulted in 8.57 million 16S rRNA gene reads with an average of 64,957 reads per sample (min = 18,882, max = 183,337). In total, 1135 unique sequence reads were identified, of which 322 could be assigned to the ‘Background bacteria’ (bacteria pre-existing in the non-axenic protist culture), while the remaining 795 ASVs were associated with the lake communities. As the focus of this study is set on the impact on enriched lake communities, sequences belonging to the background bacteria, protist chloroplasts and mitochondria were excluded from most data analyses. Furthermore, we refer to the ASVs as bacteria, due their overwhelming abundance compared to Archaea (98.46% of ASVs).

**Fig. 1:**
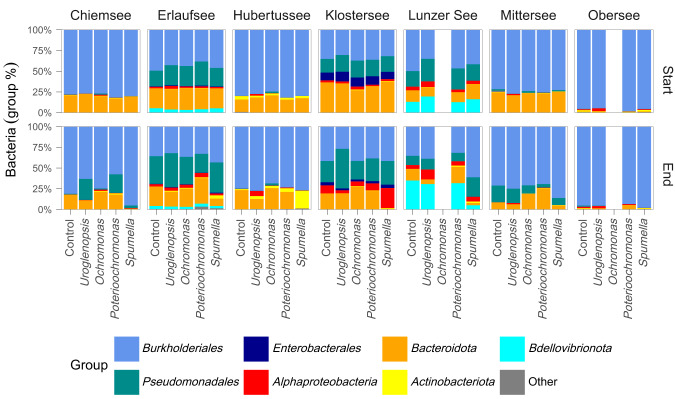
Added prey communities showed distinctive features on a broad taxonomic level depending on lake of origin. Relative abundance of bacterial groups in all treatments per experiment at the start and end of the 48 h experimental incubation are shown. At the experimental end the mean abundance of triplicate samples per treatment and lake are presented. Bacterial groups are presented at the Phylum level, except *Proteobacteria* which are split up to the class *Alphaproteobacteria* and *Gammaproteobacteria* orders (*Burkholderiales, Enterobacterales* and *Pseudomonadales*). “Other” includes diverse groups present with less than 1% abundance.

**Fig. 2:**
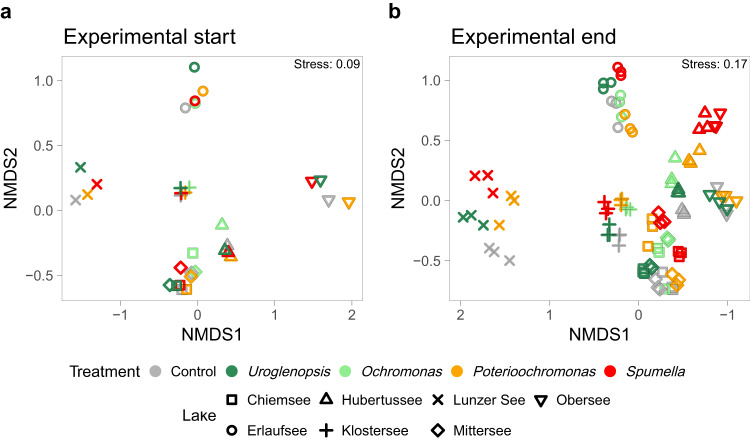
Bacterial prey communities were separated by lake of origin, yet protist impact was evident after 48 h. Added bacterial communities present at the start (**a**) and end (**b**) of all experimental incubations derived from NMDS-ordinations based on Bray-Curtis dissimilarities.

Overall, the prey bacteria prepared from the different lake inocula displayed a community composition with dominant phyla being characteristic for freshwater habitats (Fig. 1). Bacteria belonging to the order *Burkholderiales* (*Gammaproteobacteria)* were a dominant part of each lake community. Similarly, *Bacteroidota* were also abundant in almost all lakes (except Obersee). The remaining ASVs belonging to *Alphaproteobacteria*, *Bdellovibrionota, Actinobacteria, Enterobacterales* and *Pseudomonadales* (*Gammaproteobacteria)* had a high contribution only in some lakes. These differences resulted in a strong separation of start prey communities by lake (PERMANOVA R^2^ = 0.95, *p* < 0.001), with no grouping per protist treatment (PERMANOVA R^2^ = 0.05, *p* = n.s., Fig. 2a). The same separation manifests no matter if background bacteria are excluded or not from the NMDS calculations (for comparison see Supplementary Fig. S1). The start communities in Mittersee and Chiemsee overlapped, but NMDS analysis of bacterial start communities from only these two lakes showed a clear separation per lake (not shown). Thus, the goal of creating diverse bacterial prey communities differing per lake origin was achieved. After the 48 h incubation the lake origin still had a strong influence on the bacterial communities (PERMANOVA R^2^ = 0.81, *p* < 0.001), but the protists influence was also significant (PERMANOVA R^2^ = 0.11, *p* < 0.001, Fig. 2b), reflecting differential removal (due to grazing) or growth of bacteria depending on protist.

### Protist growth, bacterial growth and community turnover

**Fig. 3:**
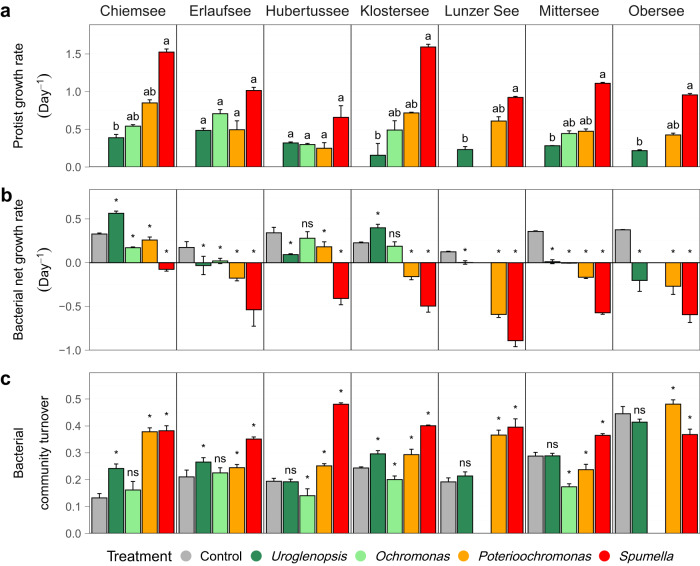
Protist growth rate (**a**), bacterial net growth rate (**b**), bacterial community composition turnover (**c**) per treatment and lake. Values are means of triplicates; error bars represent SD. Different letters indicate significant differences between the treatments per lake (Kruskal–Wallis followed by Dunn’s post hoc test, **a**). Significant and non-significant differences between the Control and a protist treatment per lake are indicated by n.s. (*p* > 0.05) and *(*p* < 0.05, Kruskal–Wallis test, **b**, **c**).

All protists attained a positive net growth during the experimental incubations (Fig. 3a). Protist growth rates differed significantly between protists consistent with their trophic mode, with the phagoheterotroph *Spumella* exhibiting the highest growth, followed by the FM *Poterioochromonas* in most incubations. The OMs *Uroglenopsis* and/or *Ochromonas* displayed comparatively lower growth rates.

The protists presence significantly affected bacterial net growth in almost all incubations (Fig. 3b). Reduced bacterial net growth in comparison to the Control, indicating grazing-induced mortality, was especially seen in the *Spumella* and *Poterioochromonas* treatments. *Uroglenopsis* incubations significantly reduced bacterial net growth in five experiments, while significantly enhancing the net growth of bacterial prey from Chiemsee and Klostersee*. Ochromonas* significantly reduced bacterial growth only in Chiemsee, Erlaufsee and Mittersee, while no significant changes were observed for Hubertussee and Klostersee.

Similar to the effects on bacterial growth, *Spumella* also had the strongest effect on BCC (Fig. 3c, see Supplementary Fig. S2 for the matching NMDS plot). In most *Spumella* and *Poterioochromonas* incubations the bacterial community turnover (change in BCC over time) was significantly higher than in the corresponding Controls. Conversely, OM displayed a stabilizing effect on BCC, with bacterial community turnover not responding much to the presence of *Uroglenopsis* and *Ochromonas*. Thereby, in *Ochromonas* BCC turnover either did not significantly differ or was reduced in comparison to the Control, and in *Uroglenopsis* a significantly higher turnover was present only in three lake experiments, without significantly differing in the remining lakes.

### Relationship between the estimated protist and bacterial community parameters

**Fig. 4:**
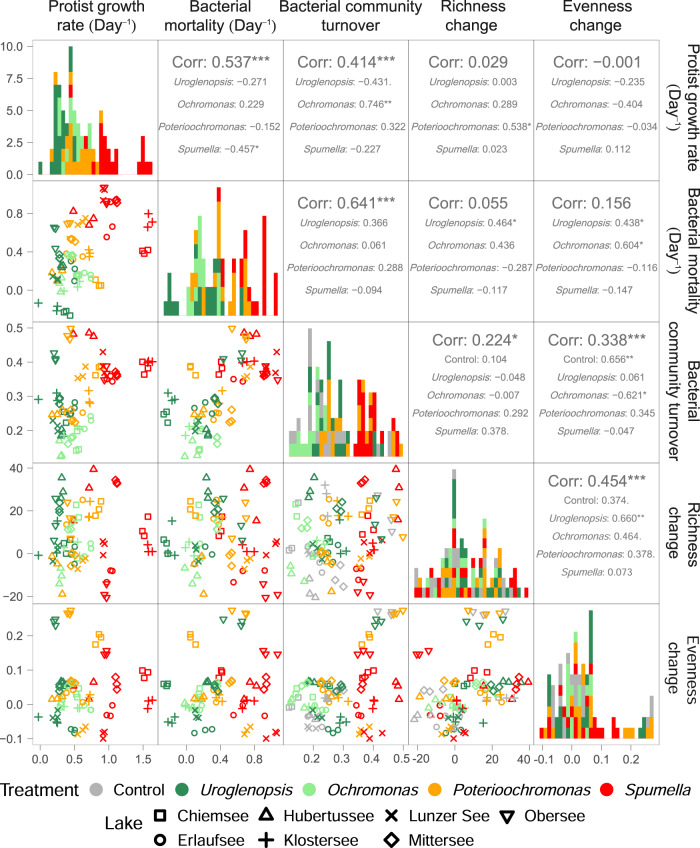
The relationship between protist growth, bacterial mortality, bacterial community composition turnover, change in species richness and evenness per treatment and lake. Spearman’s rank correlation R^2^ values are indicated by numbers, and *p* values by .(*p* < 0.1), *(*p* < 0.05), **(*p* < 0.01), ***(*p* < 0.001).

There were striking positive relationships between protist growth, bacterial mortality and community turnover throughout the experimental incubations (Fig. 4). Protist growth overall exhibited a significant positive relationship with bacterial mortality (R^2^ = 0.537, *p* < 0.001) and community turnover (R^2^ = 0.414, *p* < 0.001). Likewise, we found a significant positive relationship between bacterial mortality and community turnover (R^2^ = 0.641, *p* < 0.001). However, these trends emerging from the comparison across protist treatments did not hold within protist treatments. Only *Ochromonas* growth showed a positive relationship with bacterial community turnover (R^2^ = 0.746, *p* < 0.01), while *Spumella* growth rates and bacterial mortality negatively correlated (R^2^ = −0.457, *p* < 0.05).

The overall changes in bacterial richness and evenness did not significantly correlate with protist growth nor bacterial mortality, but exhibited a significant positive relationship with bacterial community turnover (richness change: R^2^ = 0.224, *p* < 0.05, evenness change: R^2^ = 0.338, *p* < 0.001). Trends were present for some treatments alone. *Poterioochromonas* growth positively correlated with change in bacterial richness (R^2^ = 0.538, *p* < 0.05). In the OM incubations bacterial mortality positively correlated with the change in bacterial richness (*Uroglenopsis*: R^2^ = 0.464, *p* < 0.05) and evenness (*Uroglenopsis*: R^2^ = 0.438, *p* < 0.05 and *Ochromonas*: R^2^ = 0.604, *p* < 0.05). In the Control, change in bacterial evenness exhibited a significant positive relationship with bacterial community turnover (R^2^ = 0.656, *p* < 0.01), contrary to the significant negative relationship in the *Ochromonas* treatment (R^2^ = −0.621, *p* < 0.05). Between the overall change in bacterial richness and evenness a positive relationship formed (R^2^ = 0.454, *p* < 0.001), with this relationship decreasing from primarily phototrophic towards primarily phagotrophic protist (*Uroglenopsis*: R^2^ = 0.660, *p* < 0.01, *Ochromonas:* R^2^ = 0.464, *p* < 0.1, *Poterioochromonas*: R^2^ = 0.378, *p* < 0.1, *Spumella*: R^2^ = 0.073, *p* = n.s.).

### Protist impact on specific bacterial taxa

**Fig. 5:**
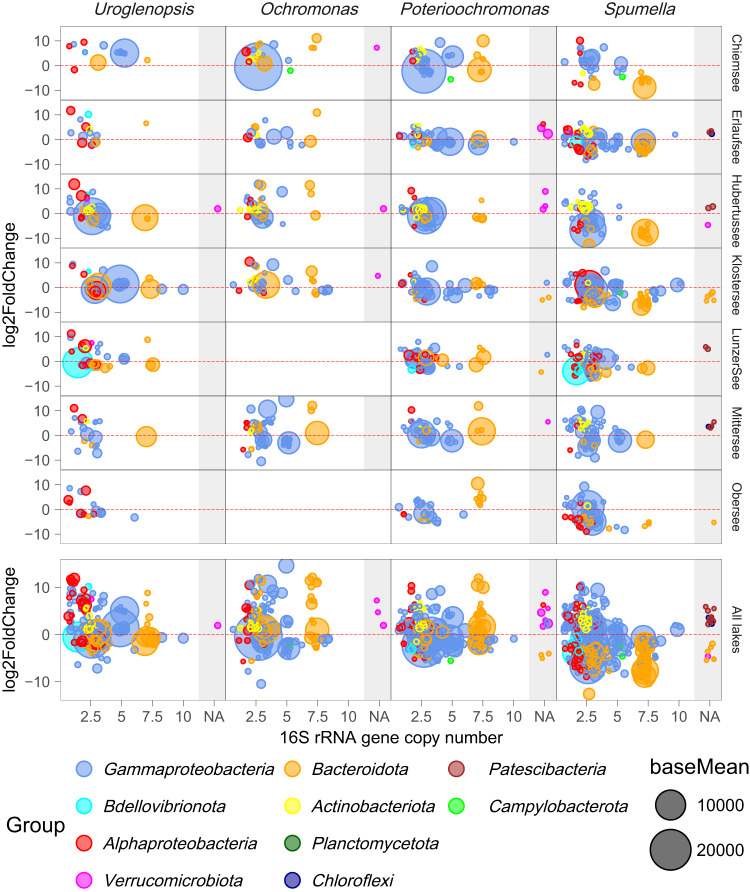
Differential abundance analysis, identifying significant responses of individual ASVs to a given protist in a given lake. Each circle represents an individual ASV. ASVs above the dotted red line are significantly overrepresented in the corresponding protist treatment and v.v. (underrepresentation likely indicating grazing). The position of each circle on the x-axis of is proportional to the rRNA Copy number of that ASV, while the area of each circle is proportional to the abundance of an ASV (baseMean, across experiment, DESeq2 R package).

**Fig. 6:**
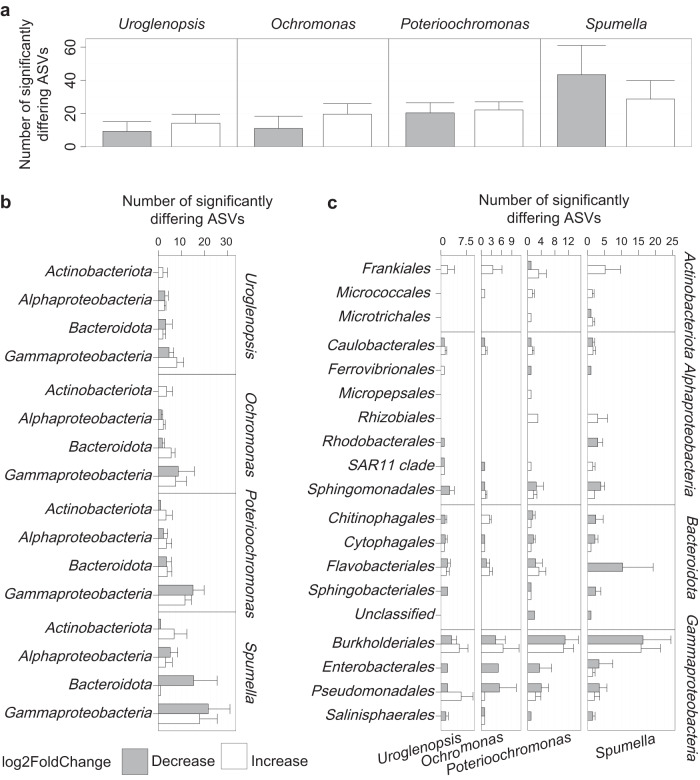
Lake-specific bacterial ASVs significantly differing between the Control and protist treatment at the end of incubation per protist. Decrease indicates potential mortality via grazing. Each barplot represents the increased and decreased ASVs of all bacterial groups combined (**a**), per bacterial phyla (**b**) and order (**c**). Values represent the mean per 7 lakes; error bars represent SD.

We tested which ASVs were significantly over- or underrepresented in a given protist incubation in comparison to the prey-only Control treatment with differential abundance analysis (Fig. 5). The strongest response in terms of ASVs showing a negative or positive response to a protist was seen in *Spumella*, with 4 and 2 times more ASVs decreasing and increasing, respectively, as compared to *Uroglenopsis*, almost 4 and 1.5 times more than in *Ochromonas*, and slightly over 2 and 1.3 times more than in *Poterioochromonas* (Fig. 6a). Thereby the average count of reduced ASVs exceeded the number of increased ASVs only in *Spumella*, while the opposite pattern was found in the mixotrophic incubations. However, the average amount of increased and decreased ASVs was almost equal in *Poterioochromonas*.

Strikingly, all protist incubations led to a significant increase of *Actinobacteria* ASVs (Fig. 6b), mainly of the order *Frankiales* (Fig. 6c, the number of significantly different ASVs at the family level is shown in Supplementary Fig. S3). In *Spumella*, ASVs belonging to *Bacteroidota* were consistently decreased during the incubation and this trend was apparent for ASVs from all orders within this phylum. *Alphaproteobacteria* and *Gammaproteobacteria* ASVs exhibited a mixed response in *Spumella* incubations, with the average count of reduced ASVs associated with these phyla exceeding the number of increased ASVs. Thereby, *Spumella* mainly affected ASVs belonging to *Flavobacteriales* and *Burkholderiales*. In *Poterioochromonas* incubations, the *Gammaproteobacteria* ASVs displayed a similar pattern to that observed in *Spumella* incubations, both at the phylum and order level. Furthermore, ASV changes at the phylum level exhibited a comparable trend in *Ochromonas* and *Poterioochromonas* incubations, with a more pronounced increase of *Bacteroidota* ASVs in *Ochromonas*. In *Uroglenopsis* incubations, the ASVs displayed a distinct response per bacterial phylum. The average amount of decreased *Gammaproteobacteria* ASVs was found to be higher than the number of increased ASVs. Conversely, *Bacteroidota* showed the opposite trend, and *Alphaproteobacteria* ASVs did not show a clear response in this context. The most affected ASVs by all three mixotrophs at the order level, i.e. *Burkholderiales* and *Pseudomonadales* ASVs did not show a uniform response per mixotroph. Even though a varying response was evident per protist treatment, shared trends were present at lower taxonomical levels (Fig. 6c, Supplementary Fig. S3). For example, all three mixotrophs consistently decreased ASVs belonging to the *Enterobacterales* (*Gammaproteobacteria*).

We could estimate 16S rRNA gene copy numbers (GCNs) on 81.26% of the bacterial ASVs (with average GCN differing between lakes, Supplementary Fig. S4a) and on 73.31% of the ASVs responsive in the DEseq2 analysis. Incubations with *Spumella* led to the biggest decrease of average GCN per lake (except in Lunzer See), while the mixotrophs did not show a clear effect (Supplementary Fig. S4b). Strikingly, in *Spumella* incubations almost all ASVs with a GCN >~5 that were significantly differentially abundant were reduced (below 0 line in Fig. 5). *Bacteroidota* with the highest GCN had a mixed response in the mixotrophic treatments, while *Gammaproteobacteria* with the highest GCN mainly got reduced in all treatments.

## Discussion

### Change in bacterial community composition was linked to protist growth and grazing rates

In accordance with our overall hypothesis, the grazing activity of the protists, measured as bacterial mortality, was linked to their impact on bacterial community composition. Community turnover (a measure of BCC change) correlated strongly with both bacterial mortality and with protist growth. In addition, protist growth correlated with bacterial mortality, an indication that bacteria were ingested and contributed to protist cell division. Thus, the observed changes in BCC were likely mainly driven by grazing activity, although other types of protist-bacteria interactions may have contributed in some cases (especially for *Uroglenopsis* and *Ochromonas*, see below). Consistent with hypothesis 1, higher grazing rates also corresponded to higher community turnover. However, the correlation between bacterial mortality and community turnover was not significant within each protist, indicating that protist-specific traits underlie this relationship. In fact, most grazing-related parameters measured formed an apparent trophic mode gradient with increasing contribution of phagotrophy (*Uroglenopsis* > *Ochromonas* > *Poterioochromonas* > *Spumella)*, which is well illustrated by the color gradient in Figs. 3 and 4.

Our experimental design does not allow us to draw general conclusions about the differential impact of mixotrophic bacterivores compared to strictly heterotrophic taxa as we only included one heterotroph (*Spumella*). However, we observed that the three mixotrophs displayed a wide range of grazing rates, both within and between experiments. *Poterioochromonas*, the facultative mixotroph (FM) induced the highest bacterial mortality of the mixotrophs on average, and also caused higher changes in BCC compared to the two obligate mixotrophs (OM). This demonstrates that the impact of mixotrophic bacterivory on BBC is far from uniform, and the specific trophic mode of the protist likely determines its impact. In some experimental incubations, the bacterial community turnover was significantly lower for the OM protists than for the prey-only Control treatment (Fig. 3c), suggesting a stabilizing effect of OM presence on BCC. This could be the outcome of their primarily phototrophic lifestyle and interactions with bacteria beyond predation. OM may have positive effects on bacteria through exudates, similar to facilitation of bacterial growth through organic exudates by photoautotrophic algae [38, 39]. This is also supported by the observation of net positive bacterial growth rates in some experiments featuring OM predators. Such interactions between mixotrophic protists and bacteria are not well studied. We do not have direct observation for phagotrophy within the two OM strains used in this study, nor for phototrophic interactions with bacteria. However, under the light and nutrient conditions in our experiment, the OM species are expected to primarily apply a phototrophic lifestyle while still ingesting bacteria [40], which was also indicated by the variable net bacterial growth rates between the experiments (Fig. 3b). Further, in order to enhance phagotrophy in the OM, we aimed to create low P concentrations within our experimental setup. An increase in phagotrophy in *Uroglenopsis* under P limited conditions has been reported (Urabe et al. 1999). In addition, the *Uroglenopsis* strain used in our study was able to achieve similarly high growth rates under P limited and nutrient replete conditions (preliminary experiments, data not shown).

### Bacterial diversity was promoted by grazing in the obligate mixotrophs

We hypothesized that bacterial diversity decreases with increased grazing rates (hypothesis 2) due to an accumulation, and resulting dominance, of grazing-resistant bacterial taxa. This expectation was not supported by our results, as there was no significant relationship between bacterial mortality and richness or evenness change. However, there was a positive correlation between bacterial community turnover and both richness and evenness change, indicating an indirect influence of grazing (bacterial mortality) on diversity via community turnover. Notably, thereby grazing would increase community turnover which in turn increases, not decreases (as hypothesized), bacterial diversity.

Within the individual protists, bacterial mortality correlated with an increase in bacterial diversity for the two OM, *Uroglenopsis* and *Ochromonas*, while such a relationship was not present for *Poterioochromonas* and *Spumella*. This suggests that grazing by these OM stimulated bacterial diversity via some mechanism. We did observe an enrichment of presumably grazing resistant bacterial taxa, e.g. belonging to the phylum *Actinobacteria* in all protist incubations (see specific phyla discussion below), yet the dominance of such taxa under high grazing rates was apparently not pronounced enough to impact bacterial richness and evenness negatively in our study. Instead, the (unselective) removal of bacterial taxa that dominated the prey communities prior to the grazing incubations, such as *Gammaproteobacteria* within the *Burkholderiales* and *Pseudomonadales*, may explain the observed increase in diversity. This mechanism, whereby predation is frequency-dependent, is central to coexistence theory in explaining maintenance of diversity [41]. *Poterioochromonas* and *Spumella* also apparently removed these dominant taxa, but at the same time also less abundant taxa. For example, the clear reduction of many *Bacteroidota* ASVs, seemingly preferably ingested by *Spumella*, indicates a strong selectivity for this prey. All in all, this is an indication that grazing by the mixotrophic protists (especially the two OM) was mainly frequency dependent, while the heterotrophic *Spumella* may employ more selective grazing mechanisms. This observation is in line with findings from another study addressing the relation between selective feeding and protist nutritional mode in marine dinoflagellates [42].

### General features of prey selectivity in bacterivorous chrysophytes

The protists employed in this study were all Chrysophytes, selected for the broad range of grazing rates, growth rates and reliance on photosynthesis that they represent. Despite the wide span in trophic modes, they are similar to each other in a number of ways. They are for example all interception feeders, which use pseudopodia to capture the prey and transport it to their food vacuoles for digestion [43, 44]. Thus, their mechanisms of selectivity may be fairly similar, even if they select for different prey.

Our experimental design allowed us to study protist-induced changes in prey community composition replicated over seven different prey communities originating from different lake ecosystems, and thereby identify general patterns in prey selectivity for the four studied protists. We observed some prey structuring patterns common to all studied protists, such as the apparent avoidance of *Actinobacteria*, which has also been observed in other studies from freshwater systems [22, 45, 46]. Most *Actinobacteria* ASVs which accumulated in the presence of the protists belonged to the order *Frankiales* and the family *Sporichthyaceae*. The family *Sporichthyaceae* is a group with a unique and complex morphology, potentially able to attach to the used incubation bottles, as they are known to have an upright posture maintained by holdfasts [47]. This, together with their thick, gram-positive cell walls and formation of chains could have led to their general avoidance by phagotrophic protists [22, 45, 46].

Another general feature common to all four protists was the apparent removal of abundant (signified by large bubble sizes in Fig. 5) ASVs belonging to the *Burkholderiales* (*Gammaproteobacteria*), specifically the family *Comamonadaceae* (Supplementary Fig. S3). This bacterial group is often dominant in freshwater ecosystems [48], with members able to outgrow other bacteria under low and enhanced predation pressure [49]. Yet, they have also been shown to be highly prone to protistan grazing [49]. In our experiment, their removal was especially pronounced in *Poterioochromonas* and *Spumella* incubations while the high growth rates of these bacteria possibly counteract the (relatively low) grazing pressure imposed by *Uroglenopsis* and *Ochromonas*. The high relative abundance of *Burkholderiales* ASVs makes them susceptible to frequency-dependent predation, due to high encounter rates. However, other mechanisms, such as cell size-based selection, and the ability to form aggregates or filaments may be equally important [50], but could not be addressed in our study.

We did not identify any clear selectivity pattern that was common to the three mixotrophic protists and not also shared by the heterotrophic *Spumella*. This indicates that there is no specific mixotrophic selectivity “fingerprint” left on bacterial prey communities via grazing, at least not for freshwater chrysophyte flagellates under the here applied conditions. On the contrary, *Spumella* displayed an apparent selectivity for *Bacteroidota* ASVs, specifically belonging to *Flavobacteriales*, unlike the mixotrophs (except *Uroglenopsis* in some experiments). Preferences for this bacterial group has been indicated previously for *Spumella sp*. and other heterotrophic species [51–53]. One striking observation is that many *Bacteroidota* ASVs had a relatively high estimated 16 S rRNA gene copy number (GCN) in their genomes, which may indicate high potential growth rates. On the one hand, fast growth rates may be a mechanism to persist in the environment, by “outgrowing” grazing pressure [54]. On the other hand, fast growing bacteria often lack grazing protective mechanisms making them suitable to grazing [53]. In addition, the high rRNA content associated to rapidly dividing cells may also make them more attractive to predators seeking phosphorous-rich prey. The apparent selection for these high GCN *Bacteroidota* ASVs by *Spumella* may therefore reflect a combination of *Spumella* being able to successfully suppress these fast-growing bacteria due to high grazing rates, and selection by *Spumella* due to food quality-related factors. It is also worth pointing out that our short experimental time may have been insufficient for certain grazing resistance mechanisms to manifest in the prey. Other studies confirmed a strong initial selection for *Bacteroidota* to shift with time towards avoidance in parallel with the formation of grazing resistant morphotypes, followed by an increased preference of *Gammaproteobacteria* subgroups [49, 55–57]. As we did not address bacterial morphology, we can only speculate that our study covered a phase where the present *Bacteroidota* still lacked effective protective mechanisms to escape the high grazing pressure of *Spumella*.

### Non-predation interactions between the studied chrysophytes and bacteria

One important observation in our study, was that the incubation of mixotrophic protists with bacterial communities led to more bacterial ASVs significantly increasing in relative abundance (compared to a prey-only Control) than those that significantly decreased. For the heterotroph *Spumella*, the opposite was true, while in *Poterioochromonas* the changes were more balanced. Generally, an increase in relative abundance during the incubation may point to an avoidance of this ASV by the bacterivorous protist, as is likely the case for *Actinobacteria*, or it could indicate a growth stimulation via the protists independent of predation activity. Alternatively, competitive release due to grazing on other bacterial taxa could also stimulate growth. Although we could not further disentangle the effects of these mechanisms with our experimental approach, it is likely that protist-induced growth stimulation played some role, especially for the mixotrophic protists. For example, the *Flavobacteriales* ASVs that were drastically removed by *Spumella* instead significantly increased in the mixotroph incubations. In the environment, *Flavobacteria* are often associated to phytoplankton blooms ([48] and references therein) and often have the ability to degrade complex polysaccharides synthesized by phytoplankton [58]. They may thus have been stimulated by the release of photosynthetic products by the mixotrophic protists in our experiment. The higher diversity of potential interactions between mixotrophic bacterivores and bacteria, including both predator-prey interactions and facilitation via different photosynthetic exudates, may thereby translate into a positive impact on the diversity of coexisting bacterial communities, which was also supported by our findings.

### Concluding remarks

By using experimental grazing incubations with diverse and variable bacterial prey communities, we aimed to elucidate general and robust top-down effects of four selected Chrysophyte flagellates on freshwater bacterial community structure. Contrary to our expectations, the heterotrophic *Spumella*, a ferocious grazer, did not affect bacterial diversity, while the mixotrophic bacterivores, specifically the two OM *Uroglenopsis* and *Ochromonas*, showed the potential to promote bacterial diversity despite moderate grazing rates. Otherwise, the three mixotrophic bacterivores did not impact bacterial community composition in any characteristic and uniform way, but rather showed indications of grazing impact being either frequency-dependent, or variable depending on protist strain. Importantly, the specific trophic mode of the protists, which was either obligate or facultative mixotrophy and pure heterotrophy, may be an equally or more important determinant of their impact on the prey than whether they were mixotrophic or heterotrophic. We highly encourage future studies focusing on protist-bacteria interactions to systematically address the influence of different protist nutritional strategies or trophic modes on these interactions, beyond the protists tested in this study.

A better understanding of mixotrophic bacterivory and its impact on bacterial communities is essential, as mixotrophs can dominate freshwater phytoplankton communities, especially in oligotrophic environments [59, 60]. Increasing evidence suggests that climate change may further enhance the importance of mixotrophic bacterivory through different mechanisms such as warming and browning [61–63]. One such example may be found in the oligotrophic lake Lunzer See in Austria, where persistent blooms of OM mixotrophs such as *Uroglenopsis* spp. and *Dinobryon* spp. were observed during long warm summers (Ptacnik, unpublished). Top-down control of bacterial populations may be particularly pronounced in oligotrophic ecosystems [64], and our results suggest that such blooming mixotrophs could also play an important role in shaping bacterial communities, even though individual grazing rates may be low compared to heterotrophic protists. We acknowledge that our experimental conditions, featuring short-term, small volume incubations under defined light and nutrient conditions, do not adequately reflect real-world freshwater ecosystems. The potential diversity-promoting effects of mixotrophic protists should therefore be further investigated during natural bloom situations or in experimental studies closely mimicking natural conditions, for example by identifying quantitatively important mixotrophic bacterivores using culture-independent tools [65, 66] and monitoring bacterial diversity. Last but not least, a mechanistic understanding of the physiology, cell biology and the genomic regulation of feeding traits for the entire spectrum of trophic modes contained within mixotrophic bacterivores is needed to fully grasp their ecological significance in changing freshwater and marine ecosystems [67–69].

### Supplementary information

Figure S1

Figure S2

Figure S3

Figure S4

Table S1

## Data Availability

Raw sequence data is available at the NCBI short read archive (bioproject PRJNA863089). All processed data will be made available for non-commercial purposes.
