# Supplementary material for: Top-down structuring of freshwater bacterial communities by mixotrophic flagellates
Source: ISME Commun. 2023 Sep 2;3:93. doi: 10.1038/s43705-023-00289-7 (PMC10475056; doi:10.1038/s43705-023-00289-7)
Supplement: Supplementary file 1 — Figure S1 [file 43705_2023_289_MOESM1_ESM.pdf]

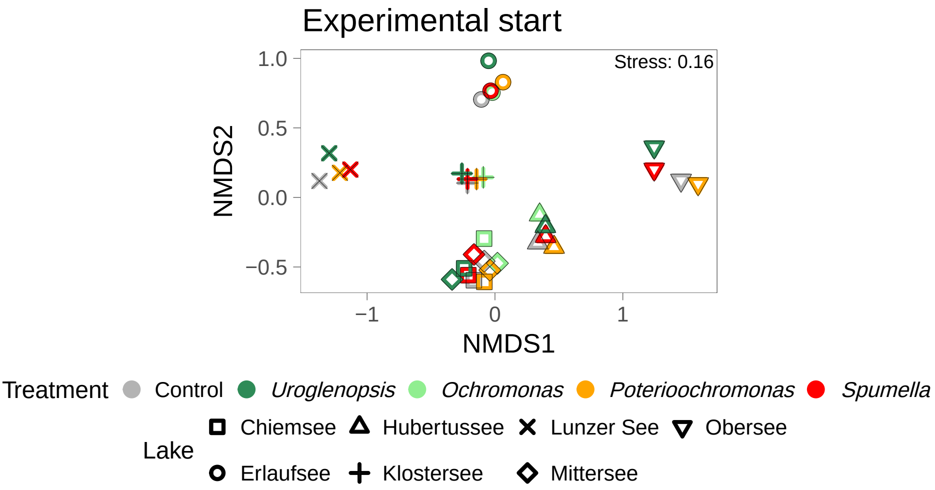

Experimental start
Stress: 0.16
NMDS2
1.0
0.5
0.0
−0.5
−1
0
1
NMDS1
Treatment
Control
Uroglenopsis
Ochromonas
Poterioochromonas
Spumella
Lake
Chiemsee
Hubertussee
Lunzer See
Obersee
Erlaufsee
Klostersee
Mittersee
