# Supplementary material for: Top-down structuring of freshwater bacterial communities by mixotrophic flagellates
Source: ISME Commun. 2023 Sep 2;3:93. doi: 10.1038/s43705-023-00289-7 (PMC10475056; doi:10.1038/s43705-023-00289-7)
Supplement: Supplementary file 2 — Figure S2 [file 43705_2023_289_MOESM2_ESM.pdf]

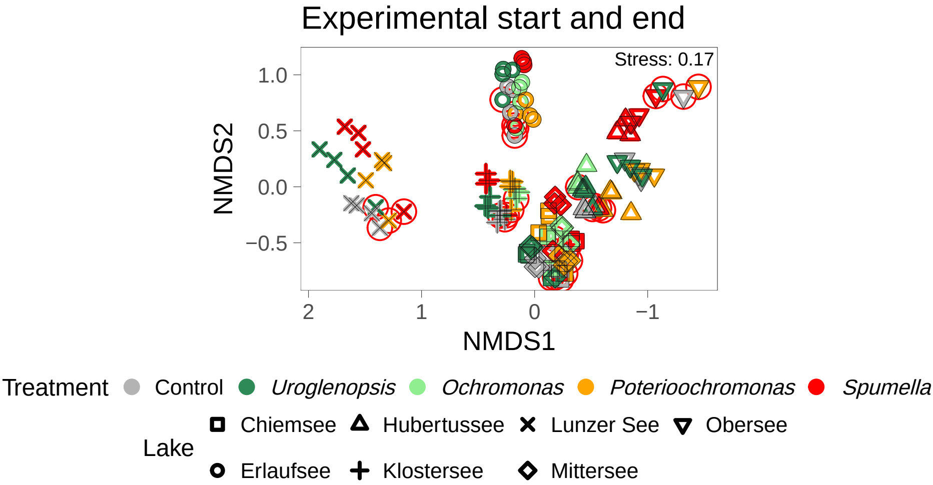

Experimental start and end
Stress: 0.17
NMDS2
1.0
0.5
0.0
−0.5
NMDS1
2
1
0
−1
Treatment
Control
Uroglenopsis
Ochromonas
Poterioochromonas
Spumella
Lake
Chiemsee
Hubertussee
Lunzer See
Obersee
Erlaufsee
Klostersee
Mittersee
