# Supplementary figures and images for: Top-down structuring of freshwater bacterial communities by mixotrophic flagellates
Source: ISME Commun. 2023 Sep 2;3:93. doi: 10.1038/s43705-023-00289-7 (PMC10475056; doi:10.1038/s43705-023-00289-7)

# Number of significantly differing ASVs

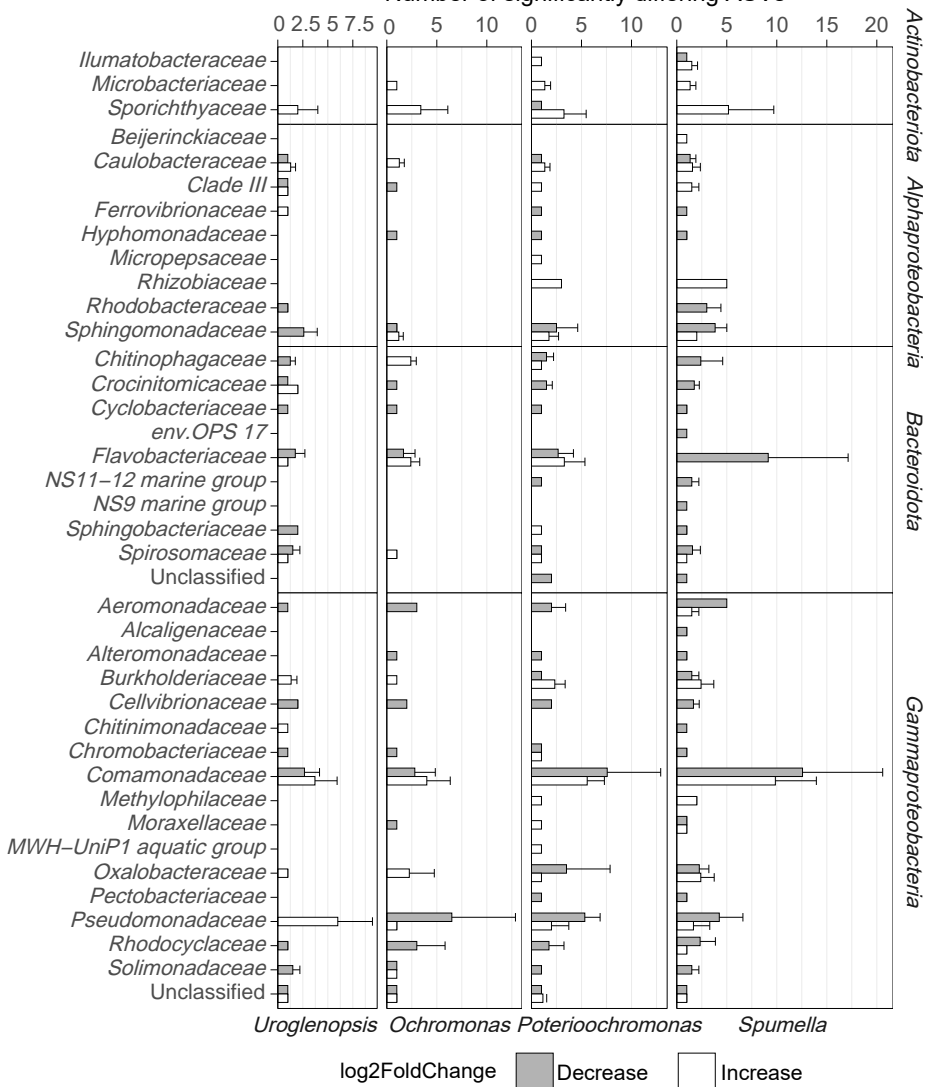

Supplement: Supplementary file 3 — Figure S3 [file 43705_2023_289_MOESM3_ESM.pdf]
