# Supplementary material for: Top-down structuring of freshwater bacterial communities by mixotrophic flagellates
Source: ISME Commun. 2023 Sep 2;3:93. doi: 10.1038/s43705-023-00289-7 (PMC10475056; doi:10.1038/s43705-023-00289-7)
Supplement: Supplementary file 4 — Figure S4 [file 43705_2023_289_MOESM4_ESM.pdf]

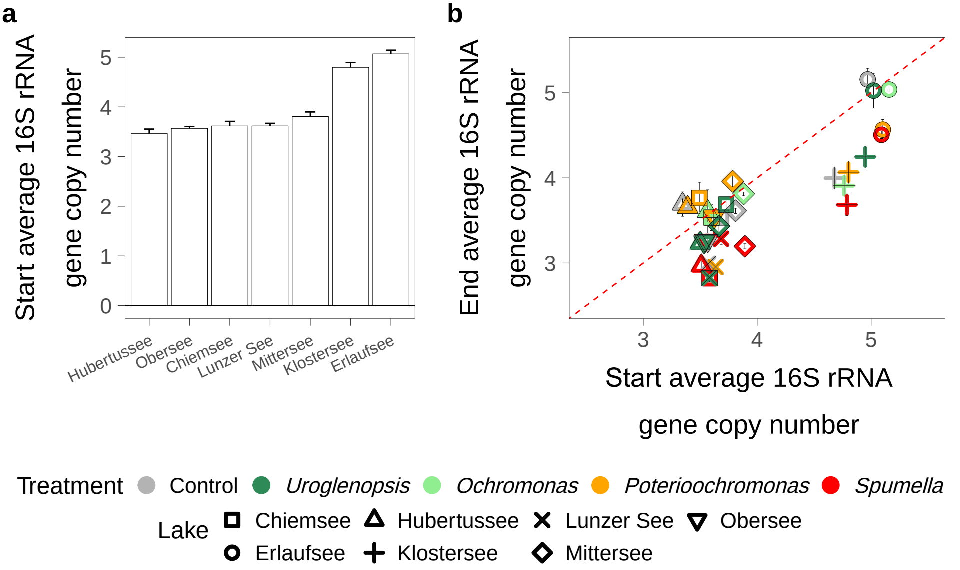
a
Start average 16S rRNA gene copy number
0
1
2
3
4
5
Hubertussee
Obersee
Chiemsee
Lunzer See
Mittersee
Klostersee
Erlaufsee
b
End average 16S rRNA gene copy number
Start average 16S rRNA gene copy number
3
4
5
Treatment
Control
Uroglenopsis
Ochromonas
Poterioochromonas
Spumella
Lake
Chiemsee
Hubertussee
Lunzer See
Obersee
Erlaufsee
Klostersee
Mittersee
